# Trichoepithelioma of the External Auditory Canal Mimicking Cholesteatoma

**DOI:** 10.7759/cureus.110301

**Published:** 2026-06-05

**Authors:** Reeba George, Katrina Minutello

**Affiliations:** 1 DeBusk College of Osteopathic Medicine, Lincoln Memorial University, Harrogate, USA; 2 Otolaryngology - Head and Neck Surgery, McLaren Oakland Hospital, Pontiac, USA

**Keywords:** adnexal tumor, bony erosion, cholesteatoma, ear canal mass, external auditory canal, histopathology, trichoepithelioma

## Abstract

Trichoepithelioma is a benign tumor arising from hair follicles that most commonly appears on the face and is rarely seen in the external auditory canal (EAC). This case describes a male aged 90 years or older who presented with purulent ear drainage and hearing loss and was found to have trichoepithelioma in the EAC. Initial imaging and clinical examination suggested cholesteatoma in the EAC with bony erosion, for which surgical intervention was indicated. However, final pathology based on more extensive tissue sampling revealed trichoepithelioma of the anterior wall of the right EAC. This case demonstrates that suspicious lesions of the EAC may require more extensive tissue sampling to prevent misdiagnosis based solely on superficial tissue samples.

## Introduction

Chronic ear disease is a common cause of outpatient otolaryngology visits [1]. Patients presenting with persistent purulent ear drainage, hearing loss, and external auditory canal (EAC) conditions raise concern for more common pathologies such as cholesteatoma, chronic otitis externa, keratosis obturans, or ear canal tumors, such as squamous cell carcinoma or basal cell carcinoma [2-4].

Cholesteatoma is a keratinizing epithelial cyst that can cause severe complications and bony destruction if left untreated [5,6]. It is a widely recognized and well-documented destructive lesion of the EAC. Surgical removal is recommended to avoid further bony erosion, infection, or major intracranial complications [5]. Trichoepithelioma, in addition, is an uncommon benign tumor originating from hair follicles that typically presents on the nose, cheeks, and eye area. Histopathologically, it is characterized by basaloid cell nests and horn cysts that may resemble other basaloid neoplasms, including basal cell carcinoma and other adnexal tumors, creating a potential for diagnostic misinterpretation. Presently, few cases are documented in the literature, making its occurrence in the EAC rare [2,7,8]. Compared to cholesteatoma, trichoepithelioma is less likely to invade bone or cause life-threatening complications, but it is more likely to be misdiagnosed due to its rarity and ambiguous clinical presentation [9,10].

This case describes an instance in which a benign tumor, such as trichoepithelioma, can mimic the more widely recognized diagnosis of EAC cholesteatoma. Improved understanding of more uncommon pathologies will enhance clinical and surgical decision-making, decrease patient morbidity, and reduce the cost of surgical and medical care [2,10].

## Case presentation

An age 90 or older male patient was referred to the neurotology clinic for evaluation of right ear pain, hearing loss, and purulent ear drainage with suspected cholesteatoma. His medical history includes myocardial infarction, mitral valve regurgitation, hypercholesterolemia, coronary atherosclerosis, chronic kidney disease, and chronic heart failure. On examination, the right anterior EAC had bony necrosis, while the tympanic membrane (TM) and middle ear were unable to be assessed due to a collapsed ear canal. He had no improvement in his hearing with hearing aids and was only able to converse with an amplifier, thus limiting daily activities.

Pure tone and speech audiometry (Figure 1) demonstrated severe to profound mixed hearing loss of the right ear and severe to profound sensorineural hearing loss of the left ear. The speech recognition threshold (SRT) of the right ear was 90 decibels (dB) and that of the left ear was 80 dB, while the word recognition score (WRS) of the right ear was 24% and that of the left ear was 16%. 

**Figure 1:**
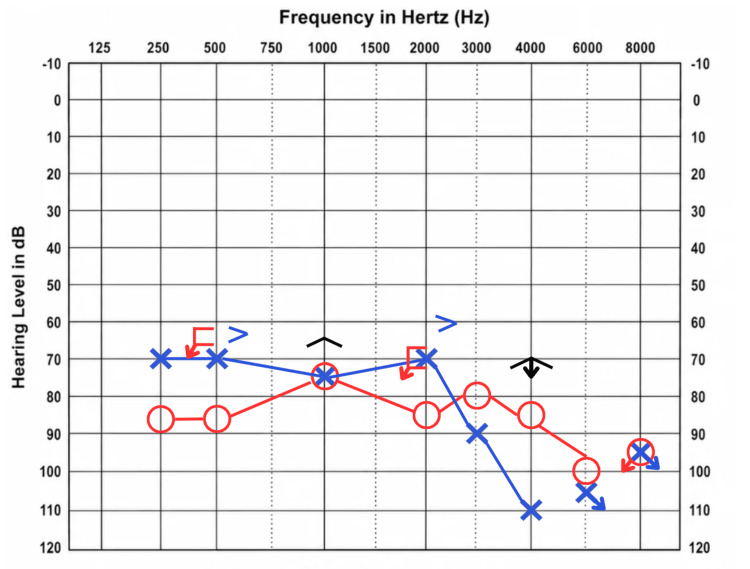
Audiogram demonstrating severe to profound mixed hearing loss of the right ear and severe to profound sensorineural hearing loss of the left ear.

A computed tomography (CT) scan of the internal auditory canals (IAC) was obtained after the initial biopsy was determined to be consistent with cholesteatoma. Imaging demonstrated complete opacification of the right ear canal and soft tissue erosion into the cortical mastoid and temporomandibular joint (TMJ), with high suspicion for EAC cholesteatoma, as shown in Figure 2. 

**Figure 2:**
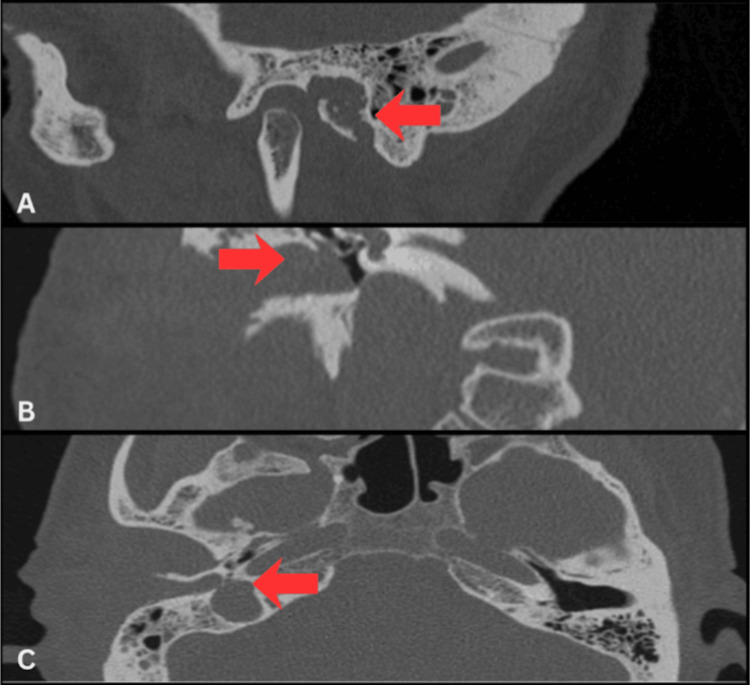
Preoperative temporal bone CT scans. (A) Sagittal view shows the right temporal bone with erosion through the mastoid anteriorly to the temporomandibular joint (arrow). (B) Coronal view shows an opacified mass occupying the entire right external auditory canal with superior and inferior distal canal erosion with intact scutum and ossicles (arrow). (C) Axial view shows opacification of the right external auditory canal and coalescence of the mastoid (arrow).

Surgical management

Surgery was indicated to remove the suspected disease, reconstruct the ear canal, and prepare for potential cochlear implantation given the patient’s significant hearing impairment and imaging findings. The diagnosis of EAC cholesteatoma was suspected based on preoperative imaging and clinical findings of bony canal erosion and inflamed, fragile soft tissue extending through the canal into the middle ear.

The patient underwent a right tympanoplasty including meatoplasty, canalplasty, and reconstruction with a pedicled middle temporal artery flap and a temporalis fascia graft. During surgery, a significant amount of fragile and scarred tissue was identified along the upper aspect of the EAC, and the ossicular chain was identified as mobile and intact. The middle ear adhesions were sent for histopathological examination.

A limited mastoidectomy was performed to ensure the diseased tissue was properly exposed and removed. A temporalis fascia graft was used to reconstruct the posterior EAC and TM, while a pedicled middle temporal artery flap was used to reconstruct the superior bony canal defect. A split-thickness skin graft from the posterior auricle was used to resurface the anterior canal wall. Finally, the canal was filled with Gelfoam soaked in ciprofloxacin-dexamethasone otic drops and stented with a nonabsorbable polyvinyl alcohol ear pack.

Final pathology unexpectedly revealed granulation tissue, chronic inflammation, and benign trichoepithelioma of the anterior canal wall.

Histopathology

The preliminary presurgical biopsies demonstrated granulation tissue and keratin debris that were consistent with an EAC cholesteatoma, with irritated squamous papilloma and keratinous material with some calcifications and signs of chronic inflammation. Special stains, including periodic acid-Schiff with diastase digestion (PAS-F) and Grocott methenamine silver (GMS), did not reveal any additional information.

During the definitive surgical excision, a number of specimens were collected for frozen and permanent histologic analysis. Intraoperative frozen section analysis of the anterior canal wall was negative for neoplasm. However, permanent sections revealed benign follicular structures composed of basaloid cells forming microscopic nests and horn cysts within a fibrous stroma, consistent with trichoepithelioma. Despite imaging and clinical findings suggestive of a destructive cholesteatoma, the absence of invasive growth on histopathology supported a benign adnexal tumor. In addition, there were patches of chronic inflammation, scar tissue, pieces of lamellar bone, and inflamed granulation tissue in the specimens taken from the skin of the right ear canal. Ulceration and reactive epithelial changes in the setting of chronic inflammation and granulation tissue likely contributed to the misleading clinical and radiologic appearance of EAC cholesteatoma. There were no signs of dysplasia or cancer.

Representative histopathologic images, as shown in Figure 3, demonstrate ulcer-associated granulation tissue, verrucous epithelial changes, abnormal follicular differentiation, and atypical misshapen hair structures, highlighting the diagnostic complexity of this case.

**Figure 3:**
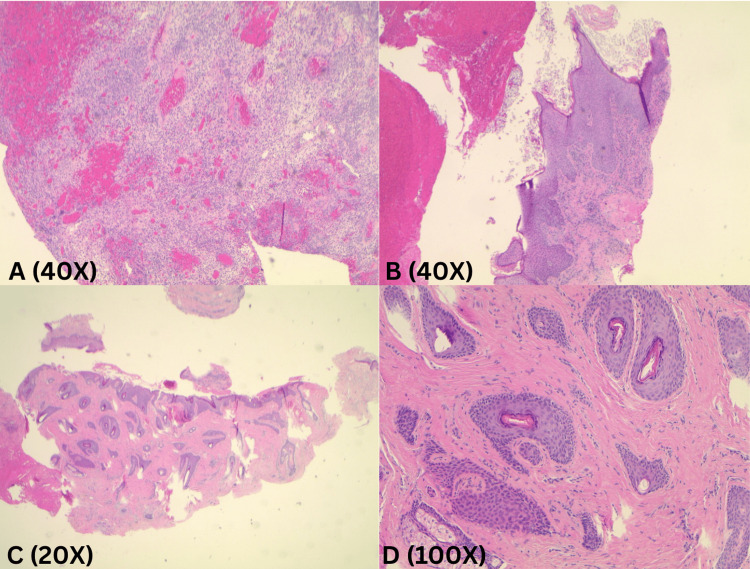
Histopathologic features of trichoepithelioma. (A) Granulation tissue with fibrovascular stroma and inflammatory infiltrate, consistent with ulceration. (B) Hyperkeratotic squamous epithelium with verruciform architecture, consistent with verruca. (C) Abnormal follicular and hair-forming structures within the dermis, consistent with trichoepithelioma. (D) Atypical misshapen hair structures with epithelial-stromal interaction, consistent with trichoepithelioma.

Trichoepithelioma is an uncommon lesion in the EAC. Despite its benign pathology, this type of tumor is usually found in facial regions with a high density of hair follicles, such as the face, rather than in the ear canal [7,9]. The histopathology of this case highlights the challenges of distinguishing among cholesteatoma, rare adnexal tumors, and chronic inflammatory processes with limited biopsy or frozen sections alone [10]. This is particularly relevant in areas of chronic inflammation and scar tissue.

The necessity of comprehensive and extensive tissue sampling in suspicious EAC lesions is highlighted by these findings [2,10]. Small or superficial biopsies may miss focal benign tumors such as trichoepithelioma, which can mimic more aggressive conditions on imaging and clinical examination.

## Discussion

Currently, there is a paucity of information regarding EAC trichoepithelioma in the broader field of otolaryngology. Magliulo et al. discussed a solitary desmoplastic trichoepithelioma of the EAC presenting as a slow-growing, painless obstructive mass without otorrhea or bony erosion on imaging [2]. Similarly, Kaluskar reported a case of multiple trichoepitheliomas involving the external ear with bilateral canal obstruction and progressive hearing loss without evidence of bony destruction, which was managed successfully with KTP/532 laser excision [8]. Both cases underwent surgical excision primarily for diagnostic purposes and without aggressive cholesteatoma surgery. In a previously reported pediatric case, otalgia and canal fullness were present without evidence of bone destruction or mastoid involvement on imaging [1]. These cases reinforce that EAC trichoepitheliomas are typically indolent, localized, and benign in nature.

This case differs from prior reports because of its aggressive clinical and imaging mimicry of an EAC cholesteatoma. The elderly patient presented with persistent purulent drainage, hearing loss, canal collapse, and bony erosion, features often associated with cholesteatoma [3,5]. To our knowledge, none of the previously reported EAC trichoepithelioma cases demonstrated this degree of chronic inflammation, bony erosion, or secondary anatomic distortion.

This discrepancy suggests that, in rare cases, secondary inflammatory changes rather than the tumor itself may lead to destructive-appearing imaging findings. Chronic infection, keratin debris, and granulation tissue can lead to pressure necrosis and reactive bone remodeling, mimicking an invasive epithelial process such as cholesteatoma [3,5]. Otologic and dermatologic literature have described cases in which benign adnexal tumors are misdiagnosed as malignant or aggressive lesions because of the surrounding inflammatory response [10,11]. Another key diagnostic challenge emphasized in this case is the limitation of superficial biopsies and frozen section analysis. Initial biopsy demonstrated keratin debris, granulation tissue, and chronic inflammation consistent with cholesteatoma. Similarly, Kang et al. reported that trichoepithelioma was misdiagnosed as basal cell carcinoma because of incomplete sampling and overlapping histologic features [10]. Only permanent sections revealed diagnostic basaloid cell nests and horn cysts characteristic of trichoepithelioma [7,12]. This further confirms observations in the head and neck pathology literature emphasizing that adnexal tumors, particularly those with a focal distribution, may be missed in limited specimens, especially in chronically inflamed tissue [5,13,14]. The risk is higher in the EAC, where inflammation, infection, and scar tissue are common.

Compared with previously published cases, this case reveals a clinically deceptive presentation of EAC trichoepithelioma mimicking cholesteatoma clinically and radiographically. This emphasizes the importance of clinicians being aware of uncommon otologic presentations when imaging findings, intraoperative appearance, and pathology are not entirely consistent. While surgical intervention was justified in this patient because of the severity of symptoms and imaging findings, this case highlights how broader awareness of rare EAC adnexal tumors can improve surgical planning and diagnostic precision when frozen section analysis is inconclusive, thereby improving patient care. In contrast, if trichoepithelioma had been suspected preoperatively, a less extensive surgical approach may have been considered, as these benign lesions typically do not require aggressive resection unless symptomatic or diagnostically uncertain. Comprehensive tissue sampling remains essential to avoid misdiagnosis and prevent unnecessarily aggressive intervention when benign pathology is present.

## Conclusions

This case describes an age 90 or older man with a rare case of trichoepithelioma of the anterior EAC wall. Due to similar clinical and imaging characteristics, the ear canal mass was initially mistaken for cholesteatoma. The case highlights the necessity of comprehensive tissue sampling, particularly in cases in which frozen sections and superficial biopsies performed during surgery led to uncertain results. This approach is essential for differentiating benign tumors from more prevalent EAC pathologies such as cholesteatoma. The case contributes to the limited literature describing EAC trichoepithelioma and demonstrates the importance of careful histopathologic evaluation to avoid misdiagnosis or excessively aggressive treatment.
